# An Integrative Pharmacology Based Analysis of Refined Liuweiwuling Against Liver Injury: A Novel Component Combination and Hepaprotective Mechanism

**DOI:** 10.3389/fphar.2021.747010

**Published:** 2021-09-22

**Authors:** Yuan Gao, Wei Shi, Hongyu Yao, Yongqiang Ai, Ruisheng Li, Zhilei Wang, Tingting Liu, Wenzhang Dai, Xiaohe Xiao, Jun Zhao, Ming Niu, Zhaofang Bai

**Affiliations:** ^1^ School of Traditional Chinese Medicine, Capital Medical University, Beijing, China; ^2^ Senior Department of Hepatology, The Fifth Medical Center of PLA General Hospital, Beijing, China; ^3^ China Military Institute of Chinese Materia, The Fifth Medical Center of PLA General Hospital, Beijing, China; ^4^ Department of Infectious Disease Medicine, The Fifth Medical Center of PLA General Hospital, Beijing, China; ^5^ Department of Poisoning Treatment, The Fifth Medical Center of PLA General Hospital, Beijing, China

**Keywords:** hepatoprotective effect, liuweiwuling, network pharmacology, component combination, antiapoptosis, anti-inflammation

## Abstract

Liver disease is a major cause of illness and death worldwide. In China, liver diseases, primarily alcoholic and nonalcoholic fatty liver disease, and viral hepatitis, affect approximately 300 million people, resulting in a major impact on the global burden of liver diseases. The use of Liuweiwuling (LWWL), a traditional Chinese medicine formula, approved by the Chinese Food and Drug Administration for decreasing aminotransferase levels induced by different liver diseases. Our previous study indicated a part of the material basis and mechanisms of LWWL in the treatment of hepatic fibrosis. However, knowledge of the materials and molecular mechanisms of LWWL in the treatment of liver diseases remains limited. Using pharmacokinetic and network pharmacology methods, this study demonstrated that the active components of LWWL were involved in the treatment mechanism against liver diseases and exerted anti-apoptosis and anti-inflammatory effects. Furthermore, esculetin, luteolin, schisandrin A and schisandrin B may play an important role by exerting anti-inflammatory and hepatoprotective effects *in vitro*. Esculeti and luteolin dose-dependently inhibited H_2_O_2_-induced cell apoptosis, and luteolin also inhibited the NF-κB signaling pathway in bone marrow-derived macrophages. schisandrin A and B inhibited the release of ROS in acetaminophen (APAP)-induced acute liver injury *in vitro*. Moreover, LWWL active ingredients protect against APAP-induced acute liver injury in mice. The four active ingredients may inhibit oxidative stress or inflammation to exert hepatoprotective effect. In conclusion, our results showed that the novel component combination of LWWL can protect against APAP-induced acute liver injury by inhibiting cell apoptosis and exerting anti-inflammatory effects.

## Introduction

Liver diseases lead to severe public health problems owing to their high prevalence worldwide and poor long-term clinical outcomes, including cirrhosis and hepatocellular carcinoma (Wang et al., 2014). Different types of liver diseases, including chronic hepatitis B virus infection, alcoholic liver disease, nonalcoholic fatty liver disease, autoimmune liver disease, and drug-induced liver disease, potentially threaten a large proportion of the global population. In China, approximately 300 million people are affected by liver diseases, which has a major impact on the global burden of liver diseases (Cui and Jia, 2013; Zhang et al., 2016; Sarin et al., 2020).

In China, many patients with liver diseases opt for traditional Chinese medicine (TCM) as an alternative or complementary therapy. In China, the use of Liuweiwuling (LWWL), a TCM compound, has been approved by the Chinese State Food and Drug Administration (CFDA) for decreasing aminotransferase levels induced by different liver diseases (Xin et al., 2009; Du and Jaeschke, 2016; Branch of Hepatobiliary Diseases, CMCA, 2020). LWWL is constituted by the following six traditional Chinese herbs: Schisandrae chinensis fructus, Fructus Ligustri Lucidi, Forsythiae fructus, Curcumae rhizoma, Perennial sow thistle, and Ganoderma spore. In particular, clinical studies have confirmed that LWWL has definite therapeutic effects on liver fibrosis (Wang et al., 2018; Li et al., 2020). The dysregulated inflammatory responses, oxidative stress and cell live/death have been widely documented as primarily involved mechanisms underlying liver diseases (Zhang and Schuppan, 2014; Li et al., 2019). Emerging evidence suggests that TCM directly regulates the production of inflammatory cytokines and chemokines to improve inflammation and liver injury. (Wang C. et al., 2016). Our previous study indicated that LWWL could attenuate hepatic fibrosis via the modulation of TGF-β1 and NF-κB signaling pathways in rat models, based on bile duct ligation (BDL)- and CCl4-induced hepatic fibrosis (Liu et al., 2018a; Liu et al., 2018b). However, it is still unclear whether the underlying mechanisms and core signaling pathways mediate the multi-linked and multi-targeted effects of LWWL against liver diseases.

Integrative pharmacology can focus on predicting potential targets, pathways, and consequences and may provide clues for designing subsequent drug studies. This study used an integrative pharmacology approach to understand the systemic, liver disease-related, and molecular effects of LWWL. Our experimental results largely validated the mechanism of action of LWWL, as predicted by the integrative pharmacology analysis.

## Materials and Methods

### Reagents and Antibodies

Dimethyl sulfoxide and ultrapure lipopolysaccharide (LPS) were purchased from Sigma-Aldrich (Munich, Germany). Apigenin, esculetin, gomisin N, schisanhenol, schisandrin A, schisandrin B, anwulignan, schisantherin A, schisantherin B, specnuezhenide, schisandrin, luteolin, quinic acid, curcumenol, and acetaminophen (APAP) were obtained from MCE (New Jersey, NJ, United States). Anti-mouse-IL-1β, anti-mouse-NLRP3, and anti-mouse-ASC antibodies were purchased from Santa Cruz Biotechnology (Beijing, China). Anti-mouse-caspase-1 p45, anti-mouse-pro-IL-1β, and anti-GAPDH antibodies were purchased from Proteintech (Chicago, IL, United States). MitoSOX was purchased from Invitrogen (Carlsbad, CA, United States).

### Preparation and Analysis of LWWL by LC-MS/MS

The formula of LWWL per dose is listed in Supplementary Table S1. Briefly, the LWWL was accurately measured 1 g in a 25 ml volumetric flask and then dissolved in methanol solution. The sample was kept at 40°C for 1 h after it was sonicated for 5min. Subsequently, the LWWL extraction was centrifuged at 4°C, 12,000 rpm for 15 min, and then the supernatant was filtered with 0.22 μm filter membrane. Finally, the sample filtrate solution was used to UHPLC analysis. To verify the metabolite components in LWWL, chromatographic analysis was performed using a Triple TOF 5600- quadrupole-LC/MS system (SCIEX Technologies, United States). The SHISEIDO CAPCELL PAK ADME column (2.1 mml.D*150 mm, 4.6 um) was performed in LC-MS/MS analysis. The flow rate was set at 0.4 ml/min and the sample injection volume was 5 μL. The mobile phase conditions were as follows: mobile phase A was 0.1% formic acid in water, and the mobile phase B was acetonitrile. The multistep linear elution gradient program was as follows: 0 → 0.5 min, 90 → 60% A; 0.5 → 4.0 min, 60 → 10% A; 4 → 9.0 min, 10 → 10% A; 9.0 → 12.01 min, 10 → 90% A; 12.01→ 15 min, 90 → 90% A. Then, A quality control sample was employed to optimize the UHPLC-Q-TOF/MS conditions. MS was performed using Triple TOF 5600- quadrupole-LC/MS with an electrospray ionization source in both positive and negative modes. The electrospray source parameters were fixed as follows: MS data were gathered in the full scan mode from m/z 50–1,250 with a scan rate of 1 spectra/s. The electrospray capillary voltage was 5.5 kV in the negative mode and 5.0 kV in the positive mode. The atomization temperature of the ion source was set to 550°C. The nebulizer pressure was set to 50 psi (GS1) and 50 psi (GS2). Air curtain gas at 35 psi and cluster voltage DP at 80 V.

### Animals

Male Sprague–Dawley rats (weight: 180–220 g) and male C57BL/6 mice (6–8-weeks old) were purchased from SPF Biotechnology Co., Ltd. (Beijing, China). All animals were maintained under 12-h light/dark conditions at 22–24°C with unrestricted access to food and water for the duration of the experiment. All animal experiments in this study were conducted according to the guidelines for care and use of laboratory animals, and the study protocol was approved by the Animal Ethics Committee of the Fifth Medical Centre, Chinese People’s Liberation Army (PLA) General Hospital (animal ethics committee approval number: IACUC-2017-003).

### Human Samples and Study Design

The study protocol was approved by the Medical Ethics Committee of the Fifth Medical Center, General Hospital of PLA (No.2015180D), registered at ClinicalTrials.gov. All volunteers in this study were self-reported as Han Chinese and provided written informed consent. Blood samples were collected from each volunteer who studied or worked at the Fifth Medical Center, General Hospital of PLA (Beijing, China). According to the overall study design, all healthy volunteers took LWWL three times per day according to the specification for 2 consecutive days, and they were prohibited from smoking and consuming alcohol, tea, and coffee drinks.

### Sample Preparation

Rat plasma, urine, liver tissue homogenate, and human plasma (500 μL) were added to acetonitrile (1,500 μL). The protein was precipitated by shaking for 1 min on a shaker. Centrifugation was performed at 13,000 rpm for 10 min. The supernatant was dried at 45°C under nitrogen. Further, 100 μL methanol:water (V/v = 1:1) was used to redissolve the samples, and 5 μL of the sample was injected for analysis.

### Network Construction and Analysis

The components detected in the blood samples from volunteers and the blood, urine, and tissue samples from animals were obtained by their CAS numbers from the open online databases TCM-SP and Chemspider. The putative targets of these compounds were predicted by PharmMapper (http://59.78.96.61/pharmmapper/) with a fit score greater than 3.0 and Z-score greater than 0. Multiple targets associated with liver diseases were collected by keyword-based searches in the Online Mendelian Inheritance in Man database; the disease names and keywords were related to the Medical Subject Headings database from the NIH and the U.S. National Library of Medicine. All collected targets were converted into UniProt IDs, and protein–protein interactions between them were screened from the Database of Interacting Proteins. Thereafter, the components and their putative targets, liver disease-related targets, and interactive proteins were combined to construct a compound–target–disease network, and Cytoscape V3.7.2 was applied to visualize and analyze the network. The topological features of each node in the network were calculated using Network Analyzer in the Cytoscape software, and the most probable disease targets on which LWWL components or their metabolites might act were screened with three topologic parameters (“Degree,” “Betweenness centrality,” and “Closeness centrality”). Only hubnodes (“Degree” greater than 2-fold of the median value) with higher “Betweenness centrality” and “Closeness centrality” (above the median value) values were identified as candidate targets of LWWL. All candidate targets were supplied to the Database for Annotation, Visualization and Integrated Discovery for Gene Ontology (GO) and pathway analysis, and only the results with Bonferroni adjustment *p*-values less than 0.01 were used for further analysis.

### Cell Culture

Bone marrow-derived macrophages (BMDMs) were isolated from the femoral bone marrow of 10-week-old female C57BL/6 mice and cultured in Dulbecco’s modified Eagle medium (DMEM) supplemented with 10% fetal bovine serum (FBS), 1% penicillin/streptomycin (P/S), and 50 ng/ml murine macrophage colony-stimulating factor. L02 and HepaG2 cells were grown in DMEM supplemented with 10% FBS and 1% P/S. All cells were cultured in a humidified 5% (v/v) CO_2_ atmosphere at 37°C.

### Cell Viability Assay

L02 cells were seeded at 8.5 × 10^4^ cells/well onto 96-well plates overnight. The cells were incubated at 37°C, followed by treatment with the active components of LWWL (apigenin, esculetin, gomisin N, schisanhenol, schisandrin A, schisandrin B, anwulignan, schisantherin A, schisantherin B, specnuezhenide, schisandrin, luteolin, quinic acid, and curcumenol) for 24 h. Thereafter, the medium was replaced with DMEM containing CCK-8 solution for 30 min. The optical density was determined at a wavelength of 450 nm.

### NF-κB Signing Pathway Activation

BMDMs were seeded at 5 × 10^5^ cells/well onto 24-well plates overnight and then treated with apigenin, esculetin, gomisin N, schisanhenol, schisandrin A, schisandrin B, anwulignan, schisantherin A, schisantherin B, specnuezhenide, schisandrin, luteolin, quinic acid, and curcumenol for 4 h. BMDMs were then stimulated with LPS (50 ng/ml) for 1 h. The proteins were then analyzed by immunoblotting.

### H_2_O_2_-Induced Hepatocyte Injury *in vitro*

L02 cells were seeded at 8.5 × 10^4^ cells/well onto 24-well plates overnight. The next day, the medium was replaced with DMEM containing apigenin, esculetin, gomisin N, schisanhenol, schisandrin A, schisandrin B, anwulignan, schisantherin A, schisantherin B, specnuezhenide, schisandrin, luteolin, quinic acid, and curcumenol; cells were incubated for 1 h and then stimulated with H_2_O_2_ for 12 h. The cell supernatants were collected, digested, and rinsed with phosphate-buffered saline. An Annexin V-FITC Apoptosis Detection Kit (BD, New York, United States) was used to detect the apoptosis of L02 cells by flow cytometry.

### APAP-Induced Hepatocyte Injury *in vitro*

HepaG2 cells were seeded at 0.25 × 10^5^ cells/well onto 24-well plates overnight. The following day, the medium was replaced with DMEM containing apigenin, esculetin, gomisin N, schisanhenol, schisandrin A, schisandrin B, anwulignan, schisantherin A, schisantherin B, specnuezhenide, schisandrin, luteolin, quinic acid, and curcumenol; cells were incubated for 1 h and then stimulated with APAP (20 mM) for 12 h. The cell supernatants were collected, digested, rinsed with Hank’s balanced salt solution, and stained with 4 μM MitoSOX red mitochondrial superoxide indicator (Invitrogen) at 37°C for 15 min. Thereafter, the cells were washed again with Hanks’ balanced salt solution and assayed by flow cytometry using a BD FACSCanto™ II cell analyzer (Franklin Lakes, NJ, United States) (Liu et al., 2021).

### Western Blotting

Protein extraction and western blotting assays on cell culture supernatant and whole cell lysis were performed as described previously (Gao et al., 2021).

### Enzyme-Linked Immunosorbent Assay

ELISA measurements of mouse IL-1β, TNF-α, and IL-6 (Dakewe, Beijing, China) levels were performed in accordance with the manufacturer’s instructions.

### Serum Biochemistry

Serum ALT, AST, DBIL, and TBA levels were determined using a commercially available assay kit (Nanjing Jiancheng Bioengineering Institute, Nanjing, China) according to the manufacturer’s instructions.

### Animal Experiments

After 7 days of adaptive breeding, mice were randomly divided into the following seven groups (n = 6): control group, APAP group (300 mg/kg), APAP + SA (schisandrin A [159.78 mg/kg]), APAP + SB (schisandrin B [162.43 mg/kg]), APAP + E (esculetin [0.93 mg/kg]), APAP + L (luteolin [8.56 mg/kg]) and APAP + SSEL group (schisandrin A [159.78 mg/kg] + schisandrin B [162.43 mg/kg] + esculetin [0.93 mg/kg] + luteolin [8.56 mg/kg]). schisandrin A, schisandrin B, esculetin, and luteolin were injected into the mice for 6 consecutive days. The normal and APAP groups were treated with the vehicle in the same manner. One hour after the final administration of schisandrin A, schisandrin B, esculetin, and luteolin, mice (except those in the control group) were administered APAP via a single intraperitoneal injection. After 12 h, the mice were euthanized, and blood samples were collected.

### Statistical Analyses

Prism 6 and SPSS statistics (version 21.0) were used for statistical analysis. All experimental data are expressed as mean ± standard deviation. A two-tailed unpaired Student’s t-test was used to evaluate significant differences between the two groups. Statistical significance was set at *p* < 0.05.

## Results

### Identification of the Active Components of LWWL in Rats and Humans

Firstly, we analysis the active components of LWWL by LC-MS/MS. As shown in Supplementary Figure S1 and Supplementary Table S1, a total of 20 active compounds were confirmed in LWWL. To examine the effective components of LWWL *in vivo*, we used UPLC/Q-TOF-MS for qualitatively analyzing the active components of LWWL in rats and humans. Rats were gavaged with LWWL (6.4 g/kg) for 2 consecutive days. After the last administration, the blood was collected from the orbital vein at 1, 2, 4, 8, 12, and 24 h. Thereafter, the serum was fully mixed after centrifugation (Supplementary Figure S2A). We also detected active components in the liver of the rats (Supplementary Figure S2B). During the animal gavage, the urine of rats was collected in a metabolic cage (Supplementary Figure S3). Subsequently, we used UPLC/Q-TOF-MS to analyze the active components of LWWL in the human body. Volunteers took LWWL for 2 consecutive days (three tablets at a time, three times per day). After the last dose, one of the volunteer’s blood was collected from the vein at 1, 2 h, the other one of the volunteers’ blood was collected from the vein at 4, 8 h, the last volunteers’ blood was collected from the vein at 12, 24 h, and the serum was then completely mixed after centrifugation (Supplementary Figure S4). Overall, as a result, apigenin, esculetin, gomisin N, schisanhenol, schisandrin A, schisandrin B, anwulignan, schisantherin A, schisantherin B, specnuezhenide, schisandrin, luteolin, quinic acid, and curcumenol were identified according to the described chromatographic conditions.

### Prediction of the Effective Targets of LWWL With Network Pharmacology

**FIGURE 1:**
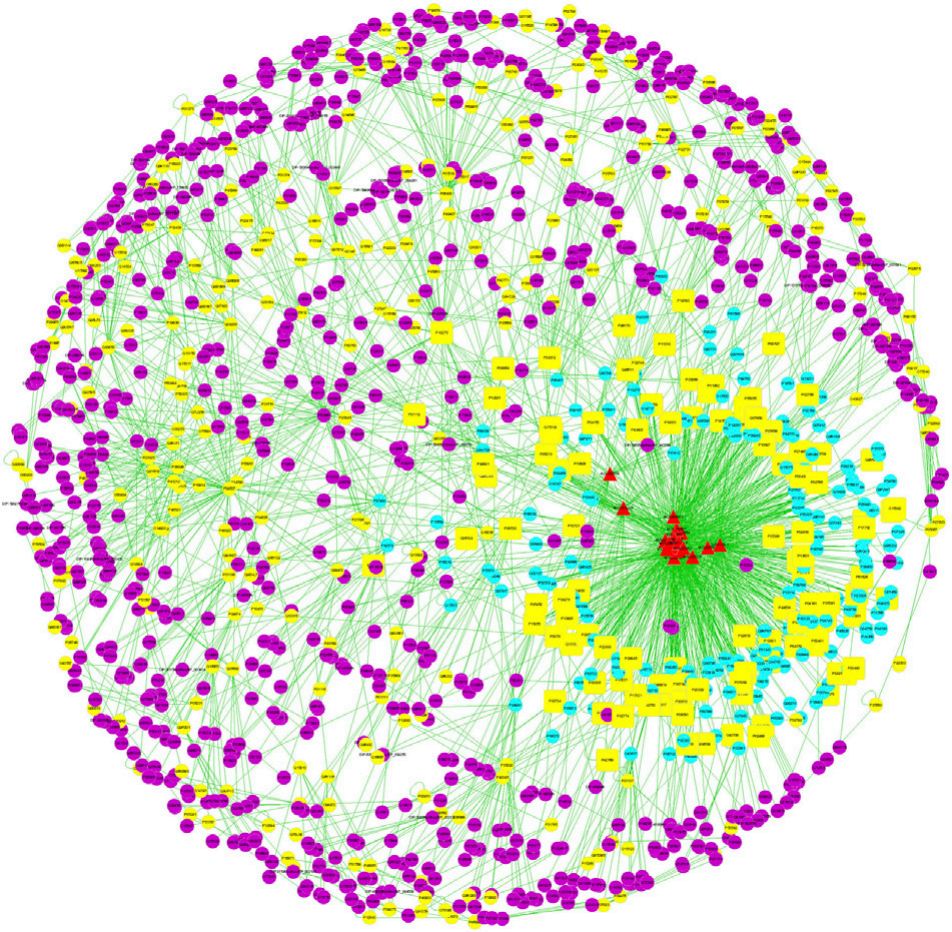
Prediction of the effective targets of LWWL with network pharmacology.

In total, 18 compounds of LWWL, 289 drug targets, 358 liver disease-related targets, and 785 interactive proteins were screened, and the network relationships between compounds and disease targets are shown in Figure 1. The topological parameters of disease nodes in the network, such as degree, betweenness centrality, and closeness centrality, were analyzed. The median values of degree, betweenness centrality, and closeness centrality were 4, 0.002, and 0.206, respectively. The hub node (>2-fold of the median degree values) with a higher betweenness centrality and closeness centrality values (more than the median values) were identified as the candidate targets of LWWL in liver diseases. As a result, 48 liver disease-related targets were identified (Table 1).

**TABLE 1:** The targets related liver diseases.

No	Uniprot IDs	Degree	Closeness	Betweenness	Direct target
			Centrality	Centrality	
1	P10275	43	0.276	0.108	Direct
2	P06213	24	0.293	0.059	Direct
3	P00533	23	0.269	0.049	Direct
4	P03372	23	0.263	0.033	Direct
5	P04150	21	0.267	0.025	Direct
6	P15056	19	0.262	0.025	Direct
7	P19793	19	0.250	0.019	Direct
8	P11362	18	0.252	0.024	Direct
9	P35968	15	0.262	0.016	Direct
10	Q02750	15	0.257	0.009	Direct
11	P12931	14	0.263	0.026	Direct
12	P31749	14	0.263	0.022	Direct
13	P01112	14	0.243	0.017	Direct
14	P00519	12	0.269	0.017	Direct
15	P02679	12	0.253	0.002	Direct
16	P49841	10	0.278	0.043	Direct
17	P13501	10	0.234	0.037	Direct
18	P08069	10	0.226	0.014	Direct
19	P35221	10	0.249	0.011	Direct
20	P20339	10	0.255	0.009	Direct
21	P55055	10	0.241	0.008	Direct
22	P22830	10	0.268	0.003	Direct
23	Q06187	10	0.236	0.002	Direct
24	P08581	9	0.252	0.006	Direct
25	P11766	9	0.266	0.002	Direct
26	P04637	60	0.285	0.147	Indirect
27	P06400	33	0.236	0.053	Indirect
28	P38398	30	0.250	0.049	Indirect
29	Q00653	28	0.220	0.017	Indirect
30	Q9Y6K9	22	0.244	0.029	Indirect
31	P35222	22	0.226	0.024	Indirect
32	P19838	21	0.221	0.013	Indirect
33	Q04206	19	0.235	0.034	Indirect
34	P01106	19	0.255	0.028	Indirect
35	Q07812	15	0.247	0.015	Indirect
36	O14920	15	0.240	0.012	Indirect
37	P05412	13	0.248	0.019	Indirect
38	Q96EB6	12	0.225	0.016	Indirect
39	Q16665	12	0.207	0.014	Indirect
40	P15692	12	0.211	0.007	Indirect
41	P09874	11	0.236	0.030	Indirect
42	P25445	11	0.229	0.017	Indirect
43	O00255	11	0.215	0.006	Indirect
44	Q09472	10	0.247	0.018	Indirect
45	O75581	10	0.224	0.011	Indirect
46	Q13315	9	0.237	0.014	Indirect
47	P27986	9	0.235	0.007	Indirect
48	Q07820	9	0.226	0.006	Indirect

**FIGURE 2:**
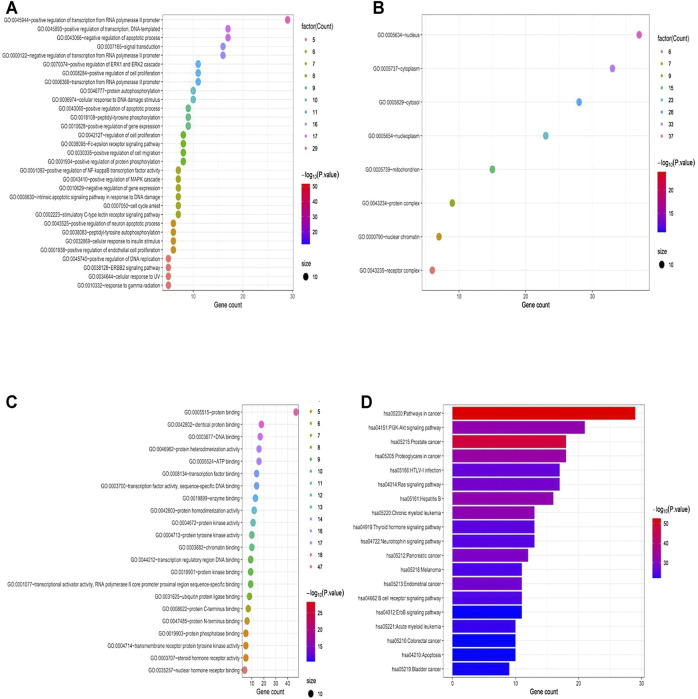
Compound–target interaction network and preliminary gene ontology (GO) analysis of drug targets. **(A)** the potential targets of LWWL mainly distributed in the cellular component, **(B)** the potential targets of LWWL can bind and activate molecular function, **(C)** the potential targets engaged in the biological process, and **(D)** KEGG pathway analysis of LWWL.

The results of GO analysis of the screened targets of LWWL are shown in Figure 2A–D. The potential targets of LWWL were mainly distributed in the cellular components of the nucleus, cytosol, cytoplasm, nucleoplasm, and mitochondria. These targets could bind and activate transcription factors, DNA, proteins, and kinases, which are mainly involved in biological processes to regulate RNA or DNA transcription, apoptotic process, ERK1 and ERK2 cascade, protein autophosphorylation, and MAPK cascade. Based on the pathway enrichment analysis, we also found that LWWL could influence many inflammation-related pathways, including the PI3K-Akt, TNF-α, MAPK, and Toll-like receptor signaling pathways. Finally, these pathways could influence the NF-κB signaling pathway. In addition, we found that the apoptosis and HIF-1 signaling pathways were influenced by LWWL. In conclusion, network pharmacology showed that the anti-inflammatory and anti-apoptotic effects of LWWL may be involved in the treatment of liver diseases.

### Effects of Active Components of LWWL on the Cell Viability

**FIGURE 3:**
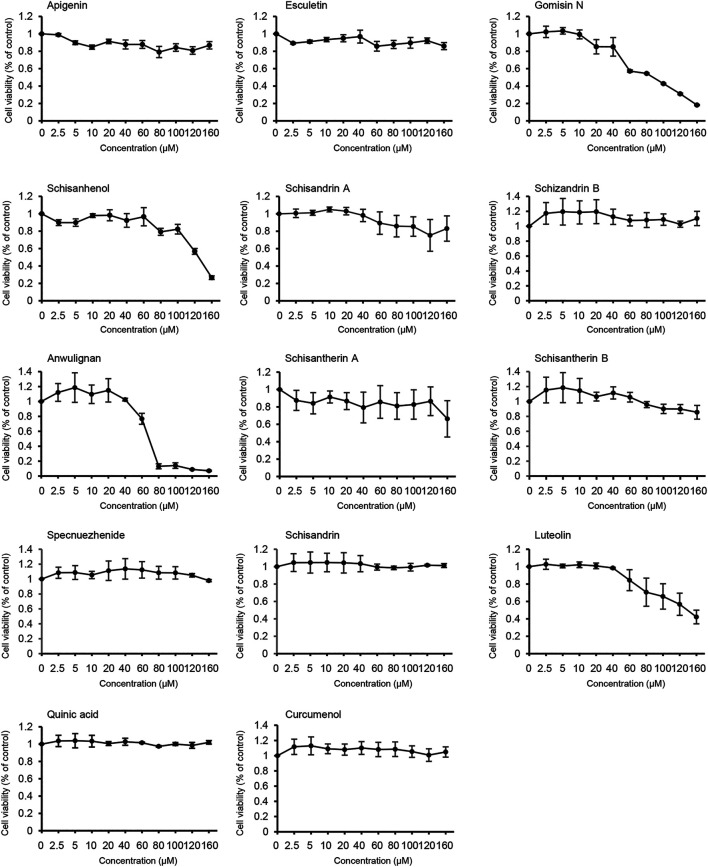
Effects of the active components of LWWL on cell viability. The viability of L02 cells treated with the active components of LWWL for 24 h was determined. Data are presented as mean ± SD using biological samples.

The effect of the active constituents of LWWL on L02 cells was tested using the CCK-8 assay. Our results showed that apigenin, esculetin, schisandrin A, schisandrin B, schisantherin A, schisandrin B, specnuezhenide, schisandrin, quinic acid, and curcumenol were not toxic to L02 cells, even at concentrations up to 160 μmol/L. However, our results also showed that after exposure to gomisin N, schisanhenol, anwulignan, or luteolin for 24 h, the half-maximal inhibitory concentration (IC50) of L02 cells was 89, 122, 68, and 123 μmol/L, respectively. However, they did not affect cell viability at low concentrations **(** Figure 3). Thus, according to our results, 0–40 μmol/L active components of LWWL were selected for the next test.

### Luteolin Inhibits NF-κB Signaling Pathway

**FIGURE 4:**
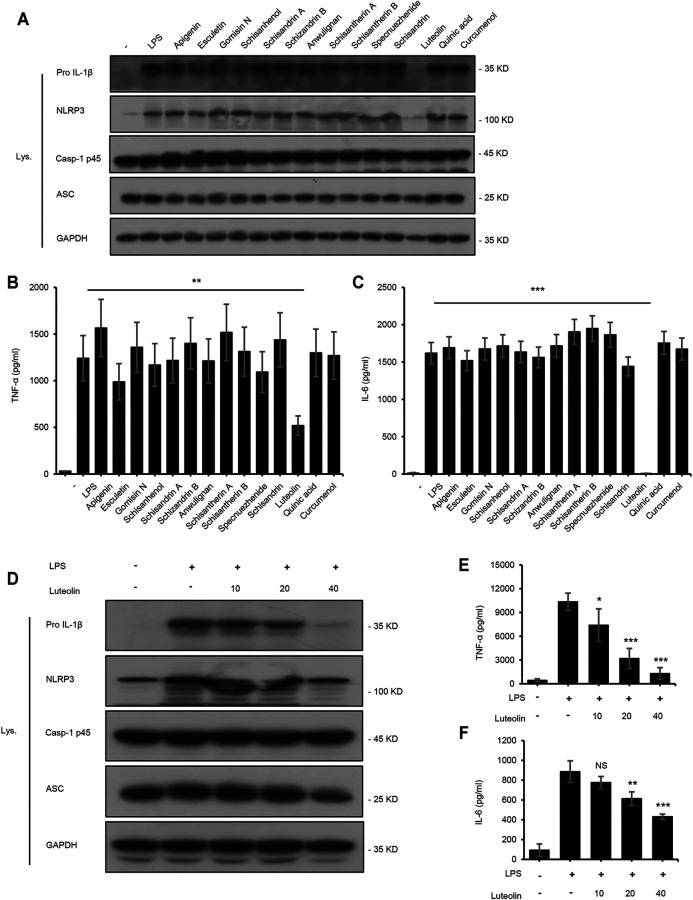
Luteolin inhibits the NF-κB signaling pathway. **(A)** Western blotting of pro IL-1β, NLRP3, Casp-1 p45, ASC, and GAPDH in BMDMs treated with apigenin, esculetin, gomisin N, schisanhenol, schisandrin A, schisandrin B, anwulignan, schisantherin A, schisandrin B, specnuezhenide, schisandrin, luteolin, quinic acid, and curcumenol (40 μM) for 4 h and then stimulated with LPS (50 ng/ml) for 1 h. **(B,C)** ELISA of TNF-α **(B)** and IL-6 **(C)** in SN from samples described in A. **(D)** Western blotting of pro IL-1β, NLRP3, Casp-1 p45, ASC, and GAPDH in BMDMs treated with luteolin for 4 h and then stimulated with LPS (50 ng/ml) for 1 h. **(E,F)** ELISA of TNF-α **(E)** and IL-6 **(F)** in SN from samples described in D. GAPDH served as a loading control. Data are represented as the mean ± SD using biological samples.

The NF-κB signaling pathway is primarily responsible for regulating the production of pro-inflammatory and pro-fibrotic cytokines and can upregulate the production of IL-1β and TNF-α(Sun, 2017). To identify the active components of LWWL that could influence the NF-κB signaling pathway, we tested the expression of TNF-α and, IL-6 and the expression of proteins related to the NF-κB signaling pathway. Our results showed that only luteolin inhibited the NF-κB signaling pathway (Figures 4A–C). To further determine the inhibitory effect of luteolin on the NF-κB signaling pathway and whether luteolin inhibited the NF-κB signaling pathway at a wide range of concentrations, BMDMs were first treated with luteolin at 0, 10, 20, and 40 μmol/L for 1 h and then stimulated with LPS for 4 h. Figures 4D–F shows that luteolin dose-dependently inhibited pro-IL-1β and NLRP3 production in cell lysates and the production of TNF-α and IL-6 in culture supernatants by ELISA assay kit. Taken together, these results demonstrate that luteolin inhibits the NF-κB signaling pathway in BMDMs *in vitro*.

### Luteolin and Esculetin Suppress H_2_O_2_-Induced Apoptosis

**FIGURE 5:**
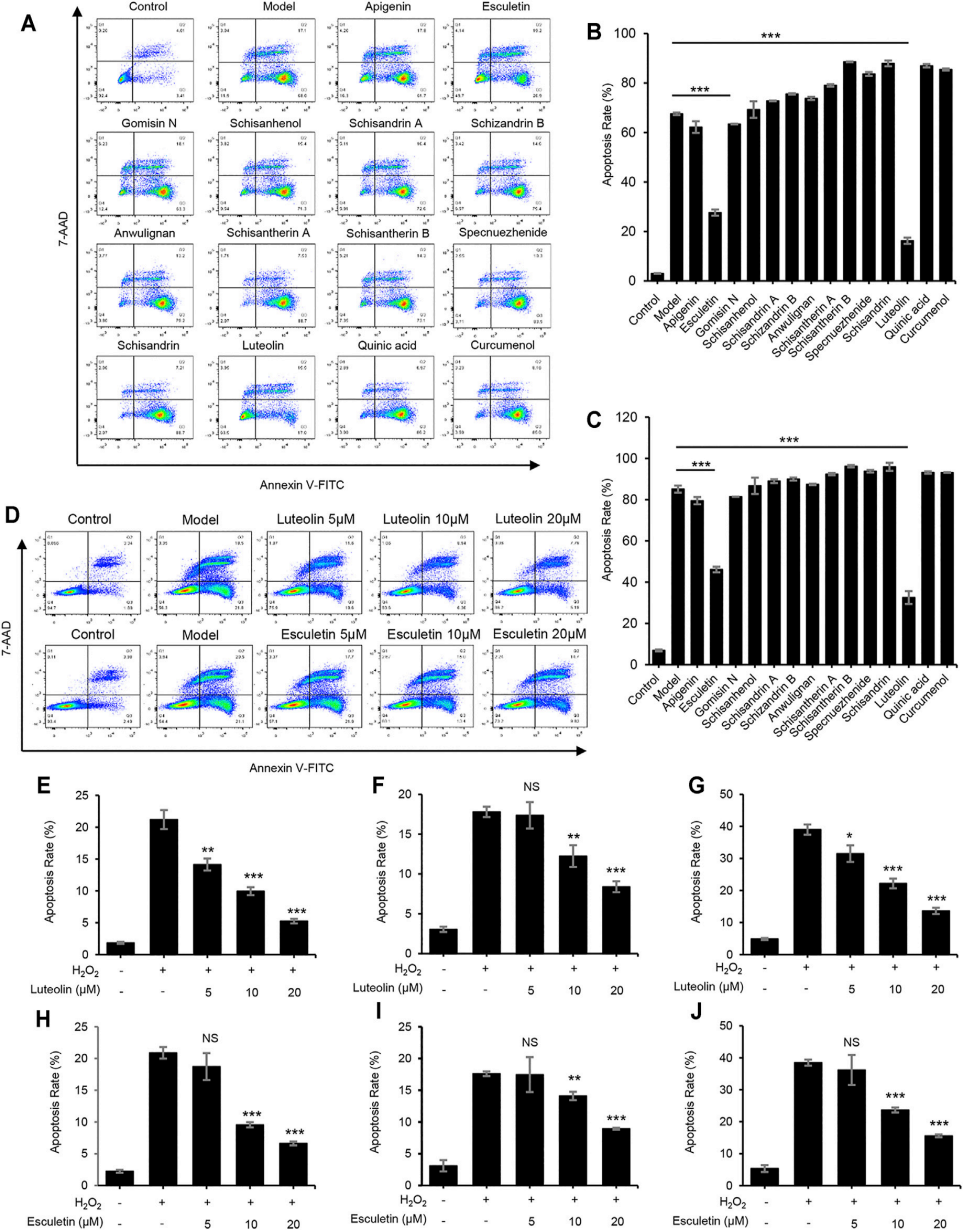
Luteolin and esculetin suppress H_2_O_2_-induced apoptosis. **(A)** Apoptosis of L02 cells treated with apigenin, esculetin, gomisin N, schisanhenol, schisandrin A, schisandrin B, anwulignan, schisantherin A, schisantherin B, specnuezhenide, schisandrin, luteolin, quinic acid, and curcumenol (40 μM) and then exposed to APAP, as detected by flow cytometry. **(B)** The percentage of early apoptotic cells from samples described in A. **(C)** The percentage of total apoptotic cells from samples described in A. **(D)** Apoptosis of L02 cells treated with esculetin or luteolin (5, 10, and 20 μM) and then exposed to APAP, as detected by flow cytometry. **(E–G)** The percentage of early apoptotic cells **(E)**, late apoptotic cells **(F)**, and total apoptotic cells **(G)** treated with luteolin (5, 10, and 20 μM). **(H–J)** The percentage of early apoptotic cells **(H)**, late apoptotic cells **(I)**, and total apoptotic cells **(J)** treated with esculetin (5, 10, and 20 μM). Data are represented as the mean ± SD using biological samples. The significance of the differences was analyzed using unpaired Student’s t-test: *p < 0.05, **p < 0.01, ***p < 0.001 vs. the control, NS, not significant.

Furthermore, the protective effects of the active constituents of LWWL on hepatocytes were examined using the Annexin V-FITC Apoptosis Detection Kit. We selected a typical hydrogen peroxide (H_2_O_2_)-induced cell apoptosis model. The results demonstrated that luteolin and esculetin treatment significantly inhibited H_2_O_2_-induced apoptosis of L02 cells. Thereafter, we treated H_2_O_2_-induced apoptosis of L02 cells with different concentrations of esculetin or luteolin. The results showed that esculetin and luteolin could dose-dependently inhibit H_2_O_2_-induced cell apoptosis at 5, 10, and 20 μmol/L, and esculetin and luteolin could also inhibit the total apoptotic cells, early apoptotic cells, and late apoptotic cells (Figure 5). These results indicated that esculetin and luteolin could protect against hepatocyte injury.

### Schisandrin A And Schisandrin B Inhibit The Release Of Reactive Oxygen Species

**FIGURE 6:**
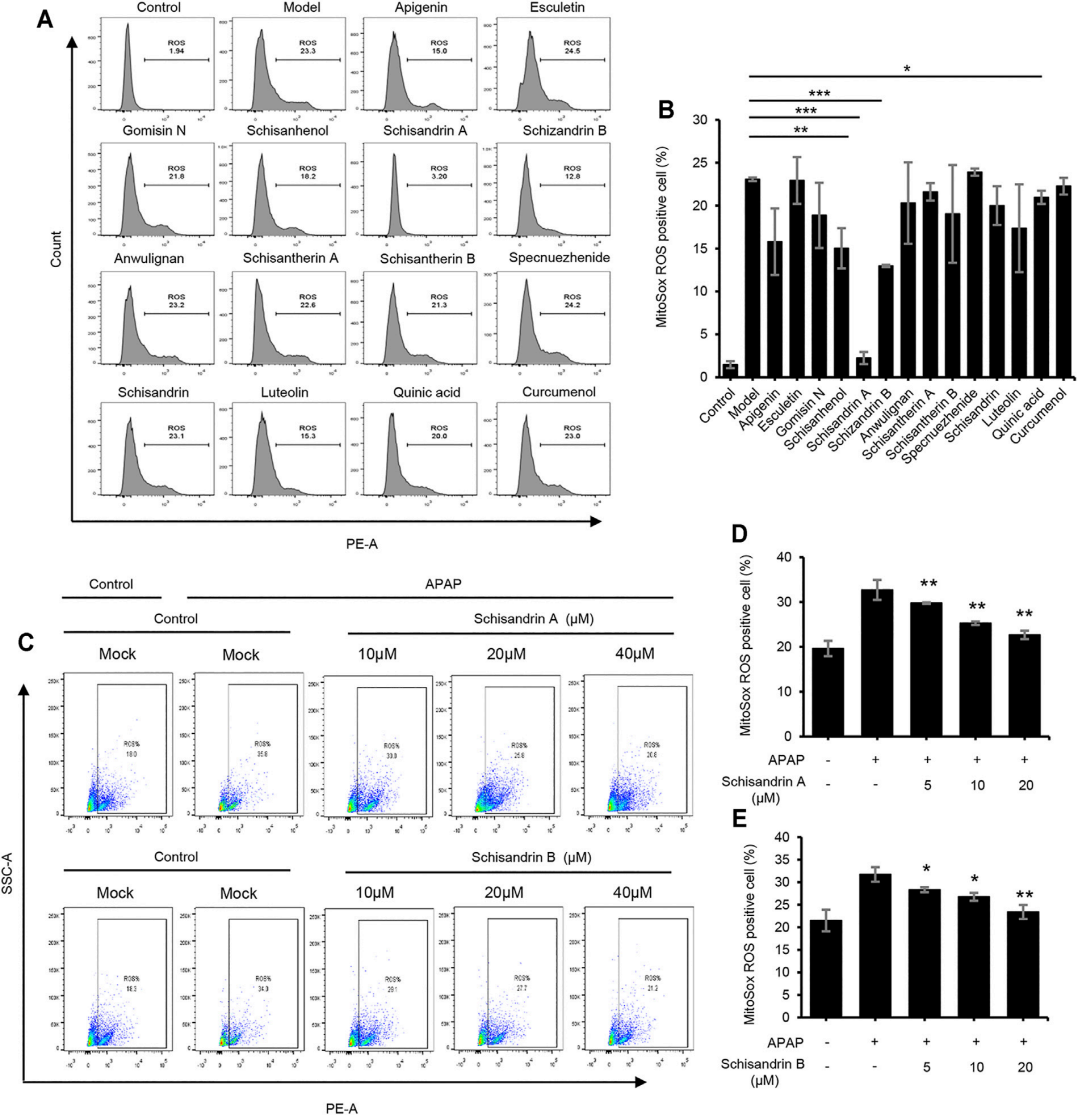
Schisandrin A and schisandrin B inhibit the release of ROS. **(A)** HepaG2 cells were treated with apigenin, esculetin, gomisin N, schisanhenol, schisandrin A, schisandrin B, anwulignan, schisantherin A, schisantherin B, specnuezhenide, schisandrin, luteolin, quinic acid, and curcumenol (40 μM) before being stimulated with APAP. HepaG2 were loaded with MitoSOX red mitochondrial superoxide indicator (Ex/Em: 510/580 nm). After staining and washing, flow cytometry was conducted to test mtROS production. **(B)** Percentage of ROS-positive cells in HepaG2 cells from samples described in A. **(C)** The production of mtROS was detected by flow cytometry in HepaG2 cells treated with schisandrin A or schisandrin B (10, 20, and 40 μM). **(D,E)** Percentage of ROS-positive cells in HepaG2 cells pretreated with schisandrin A **(D)** or schisandrin B **(E)** (10, 20, and 40 μM) and then stimulated with APAP, followed by staining with MitoSox. Data are represented as the mean ± SD using biological samples. The significance of the differences was analyzed using unpaired Student’s t-test: *p < 0.05, **p < 0.01, ***p < 0.001 vs. the control, NS, not significant.

Oxidative stress and mitochondrial dysfunction play important roles in the pathogenesis of APAP-induced acute liver injury (Miele et al., 2007). Therefore, we examined the effect of active components of LWWL on the production of ROS induced by APAP *in vitro*. Our results showed that several active components, especially schisandrin A and schisandrin B, could inhibit the release of ROS after APAP treatment (Figures 6A,B). As shown in Figures 6C–E, schisandrin A and schisandrin B dose-dependently inhibited the production of mitochondrial ROS. Therefore, schisandrin A and schisandrin B exert protective effects against liver injury by inhibiting the release of ROS *in vitro*.

### Combination of LWWL Active Ingredients Protect APAP-Induced Acute Liver Injury *in vivo*

**FIGURE 7:**
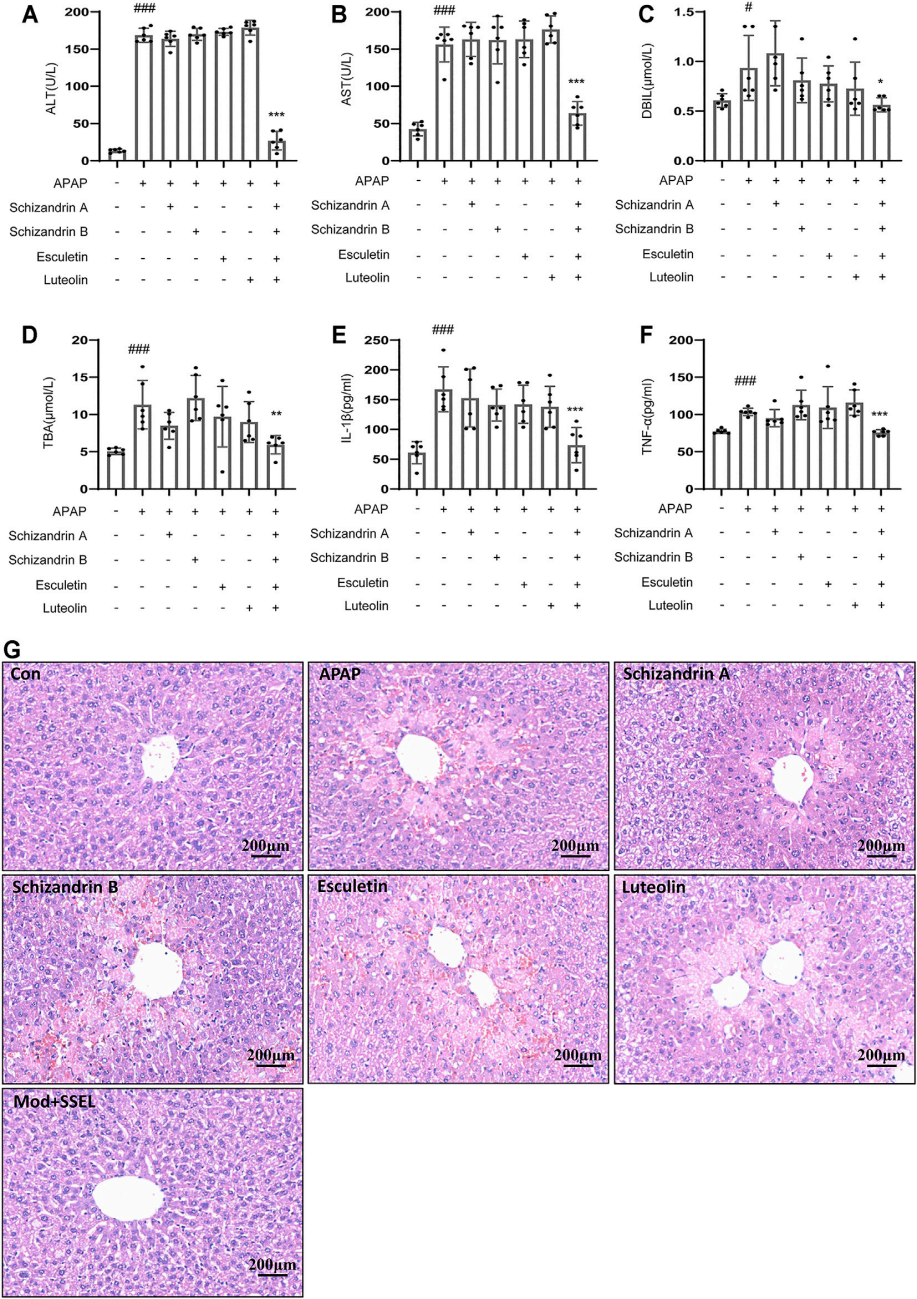
Combination of LWWL active ingredients protect against APAP-induced acute liver injury *in vivo*. **(A–G)** Eight-week-old C57BL/6 male mice were administered with a vehicle, schisandrin A, schisandrin B, esculetin, and luteolin every day by gavage for 7 days. Further, 1 h after the final gavage of schisandrin A, schisandrin B, esculetin and luteolin, mice in all groups (except the control group) were administered with APAP (300 mg/kg) by a single intraperitoneal injection. **(A–D)** Serum levels of ALT**(A)**, AST**(B)**, DBIL **(C)** and TBA **(D)**. **(E,F)** ELISA of IL-1β **(E)** and TNF-α **(F)**. **(G)** H&E staining. The significance of the differences was analyzed using unpaired Student’s t-test: #*p* < 0.05, ##*p* < 0.01, ###*p* < 0.001 vs. control group; *p < 0.05, **p < 0.01, ***p < 0.001 vs. the APAP group, NS, not significant.

To test whether the combination of LWWL active ingredients (schisandrin A, schisandrin B, esculetin and luteolin) protects against acute liver injury *in vivo*, we chose APAP to induce acute liver injury. Figures 7A–D showed that the serum levels of ALT, AST, DBIL, and TBA in the APAP-treated group were higher than those in the control group. schisandrin A, schisandrin B, esculetin and luteolin respectively treatment have no effect on APAP-induced acute liver injury. But the combination of schisandrin A, schisandrin B, esculetin and luteolin prevented the increase in serum ALT and AST levels compared with those in the APAP group. Consistent with the results of ALT and AST, treatment with the combination of schisandrin A, schisandrin B, esculetin and luteolin attenuated the expression of DBIL and TBA compared with that in the APAP group. We also detected that the combination of schisandrin A, schisandrin B, esculetin and luteolin influenced the production of IL-1β and TNF-α (Figures 7E,F). As expected, the combination of schisandrin A, schisandrin B, esculetin and luteolin treatment significantly decreased the production of IL-1β and TNF-α *in vivo*. Moreover, histopathologic studies showed that the combination of LWWL active ingredients treatment definitely alleviated the liver failure with reduced hepatocyte necrosis and liver cell degeneration (Figure 7G **)**. Collectively, these results suggest that the four LWWL active ingredients play an important role in the regulation of hepatoprotective activity.

## Discussion

At present, TCM has been confirmed to have significant therapeutic efficacy for complex diseases by exerting pharmacological effects in a multi-component and multi-target manner (Wang et al., 2018). Some Chinese patent medicines, such as LWWL and San-Cao granule, have been used clinically to treat liver diseases for many years (Wei et al., 2016). However, unclear active medicinal ingredients and mechanisms of Chinese patent medicine restrict the widespread use of TCM.

In China, LWWL, a Chinese medicine formula, is widely used to treat liver injury induced by chronic hepatitis B (Lei et al., 2015). Previous clinical and experimental studies showed that LWWL inhibits liver injury and reverses the progression of hepatic fibrosis (Liu et al., 2018a; Liu et al., 2018b). Hepatocyte injury, a primary inducer of hepatic fibrosis, can increase inflammation and activation of hepatic stellate cells (HSCs) (Ai et al., 2021). Thus, reversal of hepatocyte injury is an important way to prevent and treat hepatic fibrosis (Lee et al., 2015). Our previous studies indicated that LWWL could significantly suppress HSC activation and reverse histological fibrosis and liver injury (Liu et al., 2018b). In addition, LWWL regulates the expression of inflammatory cytokines by inhibiting the activation of NF-κB p65 and phosphorylation of IκBα (Liu et al., 2018a).

In this study, we designed an integrated strategy to explore and identify the components of LWWL by integrating network pharmacology with systems biology. We successfully employed this strategy to demonstrate the preventive effect and elementary mechanisms of LWWL against inflammatory diseases of the liver.

We used UPLC/Q-TOF-MS to analyze the active components of LWWL *in vivo*. As a result, apigenin, esculetin, gomisin N, schisanhenol, schisandrin A, schisandrin B, anwulignan, schisantherin A, schisandrin B, specnuezhenide, schisandrin, luteolin, quinic acid, and curcumin were identified. Thereafter, we predicted the effective targets of LWWL with network pharmacology. According to network pharmacology, LWWL can be used to treat liver diseases owing to its anti-inflammatory and anti-apoptotic functions.

NF-κB is one of the most important transcription factors and plays a role in the expression of pro-inflammatory genes, such as cytokines, chemokines, and adhesion molecules (Oeckinghaus and Ghosh, 2009; Aziz et al., 2018). NF-κB and upstream kinase cascades are known to have promotional roles in inflammation (Reuter et al., 2010). Inhibition of cellular inflammation has been considered a promising approach to lower the risk of inflammation-driven diseases (Rakariyatham et al., 2018). Luteolin is an abundant flavone found in LWWL, and *in vitro* and *in vivo* experiments have revealed its anti-inflammatory activity. Luteolin exerts its anti-inflammatory effects by altering the NF-κB signaling pathway (Aziz et al., 2018). In addition, cytokine regulation is crucial because cytokines are key modulators of both acute and chronic inflammation (Turner et al., 2014; Aziz et al., 2018). Moreover, luteolin significantly attenuates TNF-α-induced intracellular ROS generation (Xia et al., 2014). As expected, luteolin is an anti-inflammatory active component of LWWL and exerts its anti-inflammatory effects by inhibiting NF-κB signaling and regulating inflammatory mediators such as IL-6 and TNF-α.

Apoptosis is a biochemical process strictly controlled by an organism to scavenge dead cells through natural physiological methods (Chen et al., 2017). ROS are normal metabolites of various redox reactions in cells (Kalyanaraman et al., 2018; Gao et al., 2019). Hydrogen peroxide, the major ROS contributor in cells, is an intermediate product of oxidative metabolism in the body, affecting the structure and function of nucleic acids, membrane phospholipids, or proteins, resulting in cell damage and death (Pessayre et al., 2002). It is commonly used to evaluate antioxidant capacity, particularly for evaluating ROS scavenging capacity in cells (Wang L. et al., 2016). This study investigated whether the 14 active constituents of LWWL exert protective effects against H_2_O_2_-induced oxidative damages; we found that esculetin and luteolin could dose-dependently inhibit H_2_O_2_-induced cell apoptosis. Therefore, esculetin and luteolin are both anti-apoptosis active components of LWWL.

Some studies indicated that mitochondrial dysfunction and altered mitochondrial ROS levels affect signaling pathways, contributing to liver fibrogenesis, inflammation, and innate immune responses to viral infections (Pessayre et al., 2002; Gao and Bataller, 2011; Tell et al., 2013; Koliaki et al., 2015; Vacca et al., 2015). *Schisandra chinensis* is a commonly used traditional herbal medicine and nutritive food in many countries (Szopa et al., 2017) and has pharmacological effects in stimulating immune response and anti-inflammatory effects (Chen et al., 2019; Zhu et al., 2019). Several studies identified that the whole extract and bioactive lignans of *Schisandra chinensis* protect against liver injuries induced by hepatotoxins such as acetaminophen or carbon tetrachloride (Bi et al., 2013; Jiang et al., 2015; Fan et al., 2019). schisandrin A and schisandrin B are both the bioactive lignans isolated from *Schisandra chinensis*, which exerts hepatoprotection against liver damage (Leong et al., 2016; Zeng et al., 2017). This study also demonstrated that schisandrin A and schisandrin B could reduce APAP-induced ROS production.

In APAP-induced liver injury, oxidative stress accumulation and inflammatory responses can result in massive hepatocyte necrosis or liver failure (Paul et al., 2016). Some studies have indicated that APAP could increase the levels of lipid peroxidation products that are associated with oxidative stress, resulting in cell death or the destruction of cellular components (Li et al., 2015). After APAP overdose, cell necrosis can lead to the release of local damage-associated molecular patterns (DAMPs), which cause the transcriptional activation of inflammatory cytokines in macrophages (Williams et al., 2011; Kubes and Mehal, 2012; McGill et al., 2012; Woolbright and Jaeschke, 2017; Chao et al., 2018). Some active components of LWWL especially schisandrin A and schisandrin B could reduce the production of ROS, according to our results. Moreover, luteolin and esculetin suppressed H_2_O_2_-induced apoptosis, and luteolin, one of the active components of LWWL, could also inhibit the NF-κB signaling pathway. One of the intriguing findings in our study was that the combination of luteolin, esculetin, schisandrin A and schisandrin B decreased the serum levels of ALT, AST, DBIL, and TBA, and the combination of active ingredients significantly decreased the production of IL-1β and TNF-α. These data suggest that the combination of LWWL active ingredients could effectively protect against APAP-induced liver injury.

This study provides a better understanding of the pharmacological effects of LWWL from the perspective of its bioactive ingredients. The development of TCM formulae as a complementary and alternative therapy for liver inflammation is extremely urgent. Therefore, further development and incorporation of many disciplines (such as biochemistry, molecular biology, and bioinformatics) are necessary to elucidate the curative and biological mechanisms of TCM. The strategy proposed in this study provides a new method to identify modern indications for TCM with notable clinical efficacy using an integrated approach.

## Conclusions

In this study, we show that luteolin could inhibit the NF-κB signaling pathway in BMDMs, Esculeti and luteolin also dose-dependently suppressed H_2_O_2_-induced cell apoptosis, schisandrin A and schisandrin B inhibited the release of ROS in APAP-induced acute liver injury *in vitro*. Moreover, the combination of the four active ingredients could protect against APAP-induced acute liver injury *in vivo*. Our study indicated that combination therapy with four compounds (esculetin, luteolin, schisandrin A, and schisandrin B) inhibiting oxidative stress or inflammation exhibit better a hepatoprotective effect. In conclusion, our data demonstrate that the combination of esculetin, luteolin, schisandrin A, and schisandrin B protects liver injury by targeting different pathways, the anti-inflammatory effects of the combination of the four on liver injury is superior to that of esculetin, luteolin, schisandrin A, or schisandrin B alone. The combination of LWWL active ingredients could be applied as a potential therapy to protect or treat liver diseases in a clinical setting, but we also recognize that LWWL, as a TCM, has some limitations in its wide application in the world.

## Data Availability

The original contributions presented in the study are included in the article/Supplementary Material, further inquiries can be directed to the corresponding authors.

## Abbreviations

APAP, Acetaminophen; ALT, Alanine aminotransferase; AST, Aspartate aminotransferase; BMDMs, Bone marrow-derived macrophages; CFDA, Chinese Food and Drug Administration; DMEM, Dulbecco’s modified Eagle medium; FBS, fetal bovine serum; HSCs, Hepatic stellate cells; LWWL, Liuweiwuling; LPS, lipopolysaccharide; TCM, traditional Chinese medicine.
