# Supplementary material for: An Integrative Pharmacology Based Analysis of Refined Liuweiwuling Against Liver Injury: A Novel Component Combination and Hepaprotective Mechanism
Source: Front Pharmacol. 2021 Sep 22;12:747010. doi: 10.3389/fphar.2021.747010 (PMC8493075; doi:10.3389/fphar.2021.747010)
Supplement: Supplementary file 1 [file DataSheet1.docx]

**Table S1 Extracted ion chromatogram of compounds obtained from screening LWWL sample.**

| **Index** | **Component Name** | **RT/min** | **Adduct / Charge** | **Precursor Mass** | **Found At Mass** | **Mass Error (ppm)** |
| --- | --- | --- | --- | --- | --- | --- |
| 1 | Schisandrin A | 10 | [M+H]+ | 417.227 | 417.2279 | 1.8 |
| 2 | Schisandrin B | 10.17 | [M+H]+ | 401.196 | 401.1965 | 1.6 |
| 3 | Schisandrin C | 10.29 | [M+H]+ | 385.165 | 385.1654 | 2.1 |
| 4 | Schisandrin | 8.07 | [M+H]+ | 433.222 | 433.2232 | 2.6 |
| 5 | schisandrol B | 8.47 | [M+H]+ | 417.191 | 417.1917 | 2.3 |
| 6 | Schisantherin A | 9.36 | [M+H]+ | 537.212 | 537.2121 | 0.3 |
| 7 | Specnuezhenide | 4.67 | [M+H]+ | 687.249 | 687.2503 | 1.2 |
| 8 | Salidroside | 3.06 | [M+H]+ | 301.128 | 301.1285 | 1 |
| 9 | curcumenol | 8.83 | [M+H]+ | 237.185 | 237.1848 | -0.4 |
| 10 | Curdione | 8.29 | [M+H]+ | 237.185 | 237.185 | 0.6 |
| 11 | Germacrone | 9.25 | [M+H]+ | 219.174 | 219.1743 | 0 |
| 12 | Phillygenin | 5.5 | [M+H]+ | 535.217 | 535.2173 | -0.2 |
| 13 | Forsythiaside A | 4.3 | [M+H]+ | 625.213 | 625.2143 | 2.6 |
| 14 | Luteolin | 6 | [M+H]+ | 287.055 | 287.0553 | 0.9 |
| 15 | Ursolic acid | 10.8 | [M+H]+ | 457.368 | 457.3679 | 0.7 |
| 16 | Quercetin | 6.03 | [M+H]+ | 303.05 | 303.0504 | 1.5 |
| 17 | isoquercitrin | 4.53 | [M+H]+ | 465.103 | 465.1024 | -0.8 |
| 18 | Wogonin | 7.7 | [M+H]+ | 285.076 | 285.076 | 1 |
| 19 | Chlorogenic acid | 3.68 | [M+H]+ | 355.102 | 355.1028 | 1.3 |
| 20 | Kaempferol | 6.6 | [M+H]+ | 287.055 | 287.0554 | 1.5 |
